# Impact of Pharmacological and Non-Pharmacological Modulators on Dendritic Spines Structure and Functions in Brain

**DOI:** 10.3390/cells10123405

**Published:** 2021-12-02

**Authors:** Arehally M. Mahalakshmi, Bipul Ray, Sunanda Tuladhar, Tousif Ahmed Hediyal, Praveen Raj, Annan Gopinath Rathipriya, M. Walid Qoronfleh, Musthafa Mohamed Essa, Saravana Babu Chidambaram

**Affiliations:** 1 Department of Pharmacology, JSS College of Pharmacy, JSS Academy of Higher Education & Research, Mysuru 570015, Karnataka, India; ammahalakshmi@jssuni.edu.in (A.M.M.); bray365@gmail.com (B.R.); tuladharsunanda4@gmail.com (S.T.); tousif.a.h7@gmail.com (T.A.H.); ss.praveenraj11@gmail.com (P.R.); 2 SIG-Brain, Behaviour and Cognitive Neurosciences Research (BBRC), JSS Academy of Higher Education & Research, Mysuru 570015, Karnataka, India; 3 Centre for Experimental Pharmacology and Toxicology, Central Animal Facility, JSS Academy of Higher Education & Research, Mysuru 570015, Karnataka, India; 4 Food and Brain Research Foundation, Chennai 600094, Tamil Nadu, India; agrathipriya@gmail.com; 5 Q3CG Research Institute (QRI), Research and Policy Division, 7227 Rachel Drive, Ypsilanti, MI 48917, USA; walidq3@gmail.com; 6 Department of Food Science and Nutrition, CAMS, Sultan Qaboos University, Muscat 123, Oman; 7 Ageing and Dementia Research Group, Sultan Qaboos University, Muscat 123, Oman; 8 Biomedical Sciences Department, University of Pacific, Sacramento, CA 95211, USA

**Keywords:** dendritic spines, pharmacological modulators, modulators, yoga and meditation, enriched environment, diet

## Abstract

Dendritic spines are small, thin, hair-like protrusions found on the dendritic processes of neurons. They serve as independent compartments providing large amplitudes of Ca^2+^ signals to achieve synaptic plasticity, provide sites for newer synapses, facilitate learning and memory. One of the common and severe complication of neurodegenerative disease is cognitive impairment, which is said to be closely associated with spine pathologies viz., decreased in spine density, spine length, spine volume, spine size etc. Many treatments targeting neurological diseases have shown to improve the spine structure and distribution. However, concise data on the various modulators of dendritic spines are imperative and a need of the hour. Hence, in this review we made an attempt to consolidate the effects of various pharmacological (cholinergic, glutamatergic, GABAergic, serotonergic, adrenergic, and dopaminergic agents) and non-pharmacological modulators (dietary interventions, enriched environment, yoga and meditation) on dendritic spines structure and functions. These data suggest that both the pharmacological and non-pharmacological modulators produced significant improvement in dendritic spine structure and functions and in turn reversing the pathologies underlying neurodegeneration. Intriguingly, the non-pharmacological approaches have shown to improve intellectual performances both in preclinical and clinical platforms, but still more technology-based evidence needs to be studied. Thus, we conclude that a combination of pharmacological and non-pharmacological intervention may restore cognitive performance synergistically via improving dendritic spine number and functions in various neurological disorders.

## 1. Introduction

Small, thin, special hair-like protrusions found on the dendritic processes of neurons are known as dendritic spines. Typically, dendritic spines receive inputs from excitatory synapse in brain [1]. Spines act as independent compartments providing rapid-large-amplitude Ca^2+^ signals and are involved in achieving synaptic plasticity [2]. Based on the morphological features, spines are categorized into stubby, mushroom, thin, and filopodial spines [3]. A significant difference between them is observed across the type of neurons, age, diseased states, dendritic locations, and laminar positions [2]. Thin and stubby spines represent initial stages of spine formation while mushroom spines represent matured and stable types [4]. Spine shape influences local Ca^2+^+ concentration and cyclic adenosine monophosphate (cAMP)-regulated signaling proteins which are involved in synaptic plasticity [5,6,7]. Structural changes in spines contribute to the induction of long-term potentiation (LTP) or long-term depression (LTD), which plays a crucial role in synaptic transmissions. De novo spine growth also occurs in response to various triggers like LTP, two-photon glutamate uncaging or altered sensory experiences leading to new functional synapse formation [6,8,9,10]. LTP-induced changes in spines such as enlargement of spine head and shortening of the neck are widely evidenced from many experimental studies, explaining close association between the synaptic plasticity and spine morphology [11]. Likewise, as the magnitude of LTP induction increases in hippocampal cornu ammonis-1 (CA1) region there was a subsequent increase in the spine density, due to enhanced F-actin content, cofilin enrichment, and stabilization of F-actin content etc., [12,13,14]. Conversely, induction of long-term depression (LTD), glutamate uncaging, optogenetic stimulations reported to cause shrinkage of spine head and increased spine numbers loss [9,15,16,17,18]. Further, recent research explains the drawbacks in traditional way of dendritic spine classifications like the spine head area correlated to PSD may not, be applicable for thin and stubby spines. Hence to overcome such drawbacks a newer approach of clusterization of spines has been developed. This approach automatically groups spines into similar structural classes based on selected algorithm without a prior input. Each spine will be represented as set of value of parameters reflecting its morphology from neck and head size to complex geometrical parameters [19].

## 2. Dendritic Spines Biosynthesis

Dendritic spines exhibit a spectrum of structural reorganizations with respect to formation, maturation, and elimination, including subtle changes in size and shape modulated by neuronal activities and developmental age [20]. Current theories suggest that dendritic spines possess a chemical and electrical signaling domain that is discrete from their parent dendritic domain [21]. Most of the developing neurons form long, thin, and headless filopodia, which gets stabilized through generation of calcium transients. As filopodia are highly motile, only 10–20% of them transform to spines [22]. Filopodia formation is primarily dependent on synaptic interactions involving Rho GTPase effector P21 activated kinases (PAK). Thus, filopodia acts as precursors for spine development in the early stages [23]. Molecules like calcium/calmodulin-dependent protein kinase II (CaMKII), syndecan-2 via enabled/vasodilator-stimulated phosphoprotein (Ena/VASP) pathway [24], and paralemmin-1 [25] enhance filopodia formation and maturation of spines. While dendritic-associated adhesion molecule like telencephalin (TLCN) slows down the spine maturation by promoting filopodia formation and negatively regulating filopodia to spine transition [26]. However newly formed spines (thin and elongated) acquire post synaptic density (PSD) leading to enlargement of the head to become classical mushroom-shaped spines. Increase in the spine volume is associated with the accumulation of additional α-amino-3-hydroxy-5-methyl-4-isoxazolepropionic acid (AMPA) receptors and reorganization of actin cytoskeleton [27]. Cytoskeleton determines the shape of the spines, and actin is the major cytoskeletal protein in dendritic spines which polymerizes to filamentous actin (F-actin) through complex interactions with actin binding proteins (ABPs). Due to the polar nature, one end of actin filament grows rapidly than the other until adenosine-triphosphate (ATP) bound to the actin filament hydrolyses to adenosine-diphosphate (ADP), promoting the disassembly of pointed end by cofilin [28]. In addition to this treadmilling process complexes like actin-related proteins 2/3 (Arp2/3) induces branching of filaments [29]. The variation in the locations and turnover rates of the F-actin pools at the tip, base, and head regions of the spines contribute to the differences in the spine volume. Variations in the spine volume or enlargement is also linked to the destabilization of PSD, increased dynamics of PSD proteins like PSD-95, SHANK2, and phosphorylation of CaMKII [30]. Matrix metalloproteinases-9 (MMP-9) contributes to spine enlargement through beta-1 integrin receptor, cofilin phosphorylation, and actin polymerization. Further, the signaling pathways mediated by N-cadherin involving scaffolding proteins AF6/afadin, the Rho GTPase exchange factor Kalirin-7, Rac1, and PAKs regulate the size of spine head [31]. Rho GTPases like Rac1 or Cdc42 play important role in spine remodeling through signaling complexes including protein kinases like CaMKK and CaMKI associated with GTPase exchange factor beta-Pix or calcium/calmodulin serine protein kinase CASK [20]. Rate of elimination of spine is two-folds higher than that of spine formation between adolescence and adulthood, leading to net spine loss termed as pruning of existing connections [32]. Spine addition and elimination is related to net gain and loss of excitatory synapses over lifetime and is influenced by factors like PSD recruitment, sensory stimulation etc., [33]. Semaphorin 3F-induced activation of Tiam 1-Rac1-3-LIMK1/2-Cofilin1 and RhoA-ROCK1/2-Myosin II in dendritic spines regulates pruning of spines [34].

Pathological changes of spines are categorized into pathologies of distribution and structural pathologies [35]. Increase or decrease in the spine density [36], morphological changes viz reduction in size [15], alteration in shape [19,37], dendritic beading with loss of spines [38] and sprouting of spines (text Box 1) [39] are pathologies of distribution [40]. While structural pathologies include the changes observed with single spine like densification of cytoplasm [41,42], hypertrophy of organelles, decreased spine volume [43,44], and formation of aberrant synapse-like connections.

Alterations in spine structure and function negatively impacts learning and memory [45,46]. Majority of the neurodegenerative diseases (such as Alzheimer’s disease (ad), Parkinson’s disease (pd), Huntington’s disease (hd)) and neuropsychiatric disorders (such as depression, autism, schizophrenia,) are linked to spine dysfunction [47,48]. Mounting evidence from experimental and clinical reports indicate that the therapeutic intervention restores spine pathologies which in turn is reflected by improved learning and memory. On the other hand, non-pharmacological approaches like dietary modifications, practice of yoga and meditation, and enriched environment habitation are shown to improve memory, but very few clinical reports explain the direct effects of non-pharmacological treatments on spines structure and distribution.

Reduced synaptic plasticity, altered dendritic spine’s structure and distribution and memory impairment are key features of neurodegeneration and underlying diseases, which has been explored extensively in the past two decades. But recent evidence suggest that targeting genes, signaling molecules, and proteins involved in the biosynthesis of dendritic spines have direct effect on the synaptic plasticity and enhancement in learning and memory. Despite of extensive research there is no clear delineation on the modulators of dendritic spines, as most of the research was focused either on the disease modification or the underpinning signaling cascade. Thus, in this review, we made an attempt to summarize the effects of pharmacological (such as cholinergic agents, glutamatergic agents, GABAergic agents, serotonergic agents, adrenergic agents and dopaminergic) and non-pharmacological modulators (such as dietary modifications, enriched environment, yoga and meditation) of dendritic spines and synaptic plasticity in various conditions affecting learning and memory. An online search was conducted in PubMed, MEDLINE, Scopus, Web of Science, and Google Scholar for the published articles either experimental or reviews effecting structure and functions of dendritic spines. The data collected were then categorized into pharmacological and non-pharmacological modulators.

## 3. Effects of Pharmacological Interventions on Dendritic Spines

### 3.1. Cholinergic System Modulators

Synaptic plasticity and cognition are significantly connected to cholinergic system in the brain [49]. Genetic disruption of muscarinic and nicotinic receptors affects learning and memory [50]. Acetylcholine and other nicotinic receptor agonists are reported morphogens exhibiting potential effects on dendritic arborization [51]. In a rat model of myocardial ischemia/reperfusion (I/R) injury, a significant loss of dendritic spine is corroborated to decreased cholinergic activity [52]. Administration of donepezil, a acetyl cholinesterase inhibitor, in rats subjected to I/R injury, increased the spine numbers [53]. Similarly, administration of donepezil produced significant increase in spine numbers in prefrontal cortex pyramidal neurons of aged rats [54]. LM11A-3, a small molecule ligand for neurotrophin receptor p75 (p75NTR), reversed cholinergic neurites dystrophy, and prevented spine loss through activation of RhoA receptors [55].

Brahmi Nei, a poly herbal Siddha formulation (text Box 1), reversed cognitive decline along with increase in the dendritic length and number of mushroom-shaped spines in rodent model of scopolamine-induced dementia. The improvement in LTP is attributed to the anti-cholinesterase activity of the herbs in the formulation [56]. Phenserine tartarate (Phen), a novel acetylcholine esterase inhibitor, reversed mild traumatic brain injury (mTBI)-induced downregulation of PSD-95, and spine loss in wild type mice. Also, Phen reversed mTBI-induced loss of synaptophysin in both wild type and transgenic mouse model of AD (APP/PSEN1) [57]. Similarly, treatment with Huperzine A, a potent acetylcholinesterase inhibitor (AChEI), improved dendritic spine density and synaptotagmin levels in the cortex and hippocampus of severe model of AD (APPswe/presenilin-1) thus improved spine density is linked to reduction in amyloid plaque burden and oligomeric beta amyloid levels [58].

Manipulation of nicotinic receptors either by chrna5 deletion or by chronic exposure to nicotine during developmental stages significantly reduced apical spine density in long neurons and basal spine density in short neurons of the prefrontal L6 pyramidal neurons in mice [59]. On the similar lines, exposure of cultured hippocampal neurons to nicotine produced spine enlargement and responsiveness mediated through glutamergic transmission via α4β2 nicotinic acetylcholine receptors(nAChRs) [60]. Moreover, mice lacking β2-nAChRs showed scarcer dendritic spines when compared to the age matched wild type mice, which indicates that β2-nAChRs play a crucial role in the restoration of dendritic spine density [61]. Studies also revealed the evidence of cholinergic system modulating structural plasticity at the sub-spine level, wherein muscarinic receptor activation led to induction of fine filopodia from spine heads in the CA1 pyramidal cells of the hippocampus. The formations of spine head filopodia represents novel structural form of sub-spine plasticity which occur due to interaction between presynaptic buttons and dendritic spines [62]. Overall, increase in both basal and apical spine numbers, increased spine density, enhanced dendritic length, alterations at sub-spine level (formation of fine filopodia), and increased synaptotagmine levels in various brain regions are regulated by the cholinergic system. Thus, cholinergic system either through activation of muscarinic or nicotinic receptors or through inhibition of acetyl choline esterase makes significant impact on the formation and functions of dendritic spines.

### 3.2. Effect of Glutamatergic Modulators on Dendritic Spines

Glutamate being an important excitatory neurotransmitter in the central nervous system plays a crucial role in learning and memory and synaptic plasticity. Researchers have demonstrated that the dendritic spines also receive the glutamergic input directly from the presynaptic terminals. A study demonstrated that, application of shorter pulses of glutamate resulted in elongation of spines while long pulses led to shrinkage of the same set of spines. This explains that the differential responses exerted by glutamate depends mainly on the intensity and duration of application. [63]. Calcium released from internal stores significantly changes the spine shape of the dendritic spines (elongation of spine neck) in cultured hippocampal neurons [64]. Activation of group 1 metabotropic glutamate receptors (mGLUR1) induces release of calcium from internal stores triggering dendritic protein synthesis, thereby contributing to increased spine length [65]. Furthermore, increase in the spine length and sprouting of spines is correlated to increased synaptic activation of N-methyl-D-aspartate (NMDA)-glutamate receptors [66]. In contrast, NMDA receptor overactivation also results in spine retraction [67]. Spines formed initially in response to NMDA receptor activation are further stabilized through activation of α-amino-3-hydroxy-5-methyl-4-isoxazolepropionic acid (AMPA) receptors. Activation of AMPA receptors blocks further growth of spines and helps stabilizing spines through post synaptic membrane depolarization and calcium influx via voltage-activated calcium channels [68]. Multiple reports demonstrate the beneficial role of glutamate and its modulators in achieving growth and maintenance of the dendritic spines and its alteration in few pathological conditions. Ageing-related cognitive decline is attributed to diminished spine clustering and density in the CA1 region of hippocampus. Treatment with riluzole, a presynaptic NMDA receptor antagonist, increased thin spine density and non-linear thin spine clustering by increasing the glutamate uptake via glial transporters (EAAC1) and thus increases the synaptic glutamergic activity [69]. Moreover, positive (3-cyano-N-(1,3-diphenyl-1H-pyrazol-5-yl) benzamide) and negative (1-(3-chlorophenyl)-3-(3-methyl-5-oxo-4Himidazol-2-yl) urea) allosteric modulators of mGLuR5, differentially increased spine density in the pyramidal cells of medial prefrontal cortex [70]. Similarly, Gamma-aminobutyric acid (GABAergic) striatal spiny projection neurons co-cultured with glutamatergic cortical neurons showed increased LTP, spine numbers, and GluA1 cluster densities on NMDA activation. These data demonstrate the necessity of NMDA receptor activation to drive glutamatergic structural plasticity in striatal spiny projections [71].

Ketamine, an NMDA receptor antagonist, improved dendritic spine numbers by enhancing Ca^2+^ levels in dendritic spines of prefrontal cortex [72]. LY341495, a group II metabotropic glutamate 2/3 receptor (mGlu2/3) antagonist, acts at mammalian target of rapamycin complex 1 (mTORC1) and AMPA receptors, restored dexamethasone-induced decrease in dendritic outgrowth and spine density [73]. Thus, from the above reports it is implicated that, calcium-driven activation of NMDA-glutamate receptors and glutamate agonists is shown to increase spine length, sprouting of spines, spine density, spine clustering, and dendritic outgrowth.

### 3.3. Effect of GABAergic Agents on Dendritic Spines

GABA, a major inhibitory neurotransmitter, causes membrane hyperpolarization in adult neurons through opening of chloride (Cl−) channels. However, during embryonic development GABA receptor activation in the motor neurons of the spinal cord causes membrane depolarization due to increased intracellular concentration of CI−, mediated through a bumetanide-sensitive Na+/K+/2CI− cotransporter (NKCC1). Disruption of GABA receptor activation by bicuculline in vivo (chicken embryos) significantly reduced the dendritic outgrowth in terms of change in the number of branch points and number of ends at different dendritic orders [74]. Pharmacological blockade of GABAA receptors resulted in profound increase in the elimination of pre-existing spines persisting for first 4 postnatal months, without affecting new spine formation, also, resulted in structural stability of mature circuits. This demonstrates the role of GABAA receptors in preventing over-pruning of spines during spine refinement in the mouse cortex [75]. Thus, maturation of dendritic morphology and modulation of spine turnover in developing neurons is attributed to the excitatory GABA-driven activity. However, lowering the concentration of intracellular CI− (due to lowered expression of chloride exporter KCC2) shifts the excitatory effect of GABA to inhibitory effect in matured neurons. The switch in GABAA transmission from developmental depolarizing to hyperpolarizing was recapitulated in organotypic hippocampal slice cultures.

Studies have demonstrated that the application of GABAA antagonists to organotypic hippocampal neuronal cultures showed marked loss of spines. Upon administration of bicuculline, a GABAA receptor antagonist decreased spine density, explains that absence of hyperpolarizing GABAA transmission resulted in the spine loss [76]. Activation of GABA receptors suppressed overall cytosolic calcium concentration promoting spine shrinkage and elimination in CA1 region of rat hippocampus. However, observed local spread of spine shrinkage was attributed to induction of calcineurin and actin depolymerizing factor, i.e., ADF/cofilin (dephosphorylated) signaling cascade [16]. Mutations in alpha 1 subunit of GABAA receptor, specifically GABRA1 missense mutation (alpha1- A322D) resulted in significant increase in the number of mushroom-like spines and spine density in pyramidal cells. A322D expression on the GABAergic cells increased perisomatic bouton density [77]. Activation of GABAB receptors in a single dendritic spine of layer 5 pyramidal neurons inhibited NMDA receptors, said to be mediated through downregulation of cAMP and PKA, explaining the mechanism of establishment of neuromodulatory microdomains in the subcellular compartments like dendritic spines [78]. Blockade of GABA synthesis in cultured hippocampal neurons by mercaptopropionic acid resulted in a significant increase in the dendritic spine density. It is also proved that stressful experience promotes contextual fear memory and enhanced spine density, however administration of midazolam, a positive modulator of GABAA sites, prevented the influence of stress on both fear retention and hippocampal dendritic spine modelling in the basolateral amygdala complex (BLA) and dorsal hippocampus [79]. Together these studies demonstrate that the GABAergic agents modulate dendritic spine structure and functions differentially during embryonic development and in adult neurons.

### 3.4. Effect of Serotonergic Agents on Dendritic Spines

**Figure 1:**
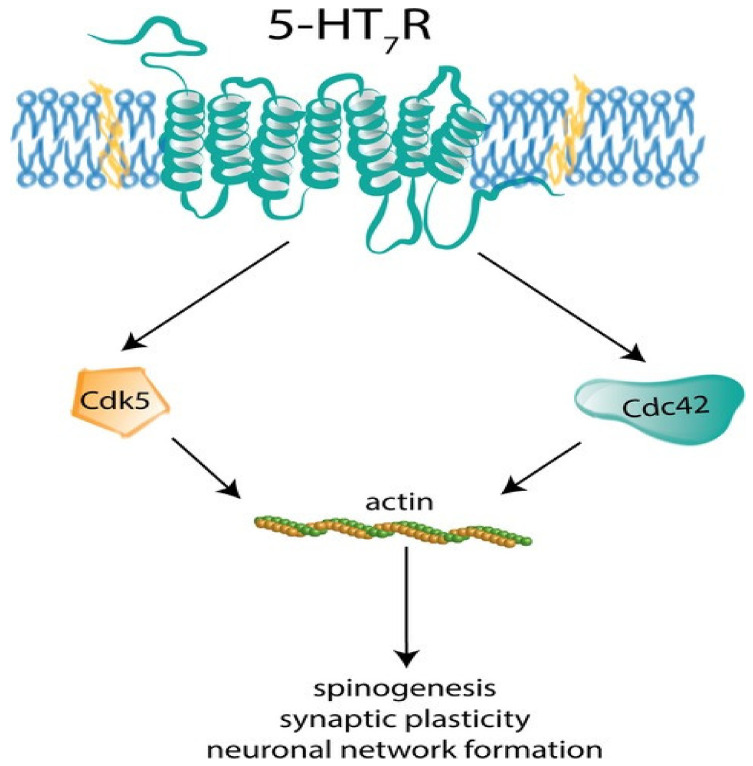
5-HT7R activates downstream small molecule GTPase’s Cdk5 and Cdc42 leading to actin polymerization and spinogenesis [89] (reused as per the *Journal of Neurochemistry* copyright permission policy) (CdK5: Cyclin Dependent Kinase5,Cdc42: Cell Division Control Protein 42).

Several studies have indicated that 5-hydroxy tryptamine (5-HT) is synthesized in the fetus early in the embryonic development [80]. In addition to the endogenous 5-HT, the brain of the fetus also receives 5-HT from the mother through placenta, emphasizing its significance in the early embryonic development including brain [81]. Establishment of cortical circuits, neuronal migration, and dendritic differentiation are few of the crucial cellular processes mediated by 5-HT system [82]. In mammalian brain, serotonergic neurons execute their functions through 20 subtypes of receptors belonging to 7 subclasses. Stimulation of 5-HT7R/Gα12 signaling pathway in one-month-old mice hippocampus has shown to increase the formation of dendritic spines [83]. In addition to the role of 5-HT in the embryonic and early post-natal life, experiments performed on adult mice by administering LP-211, a potent 5-HT7 agonist, showed significant increase in the total number and density of dendritic spines in striatal neurons. Binding of LP-211 to 5-HT7R activates downstream small GTPases like Cdk5 and Cdc42 leading to actin polymerization in turn spinogenesis (Figure 1) [84]. It was also found that 5-HT7R stimulation enhances local matrix metallopeptidase-9 (MMP-9) activity in the mouse brain, triggering dendritic spine remodeling and synaptic pruning, leading to the cleavage of CD44 followed by Cdc42 activation [85]. In an experimental model of neuroblastoma cells, activation of 5-HT4R boosts phosphorylation of cofilin and maturation of dendritic spines via RhoA-dependent control of F-actin. It was found that postnatal expression of 5-HT4R and 5-HT7R in hippocampus is differently regulated. In the early postnatal stages, 5-HT7R activation is responsible for arborization of dendritic trees and spinogenesis, while in the later developmental stages, 5-HT4R is involved in the maturation and stabilization of spines [83]. Exposure of 5-HT2 agonist, DOI to cultured cortical neurons induced transient increase in dendritic spine size as well as spine remodeling by phosphorylating PAK, a downstream target of neuronal Rac guanine nucleotide exchange factor (RacGEF) kalirin-7 [86]. Studies have also shown that short-term regulation of dendritic spines occurs through acute stimulation of 5-HT2A/2C receptors by activating phospholipase-C (PLC) which induces downstream Ca^2+^-dependent TGase activation catalyzing G proteins of Rho family and thus, activating Rac1 and Cdc42. Rac1 and Cdc42 in turn regulate the dynamics of actin cytoskeleton leading to transient dendritic spine enlargement, and thus, contributing to spine plasticity. But chronic treatment with olanzapine, 5-HT2A receptor antagonist, increased expression of Nrg1 gene, which promotes spine maturation via JAK2/STAT3 pathway [87]. Vortioxetine, agonist at 5-HT1A and 5-HT1B receptors induced spine enlargement contributing to more functional synaptic contacts in rat hippocampus [88].

### 3.5. Effect of Adrenergic Agents on Dendritic Spines

Noradrenergic system influences multiple brain functions like arousal, perception, attention, learning, and memory by regulating synaptic plasticity and dendritic spine dynamics via the modulation of cell surface receptors (NMDARs, AMPARs), protein kinases, and phosphatases [90,91]. Noradrenergic agonists are known to elevate intracellular cAMP concentration, tropomyosin receptor kinase-B (TrKB) phosphorylation and its translocation to spines thus enabling TrKB interaction with PSD-95 [92]. Studies report that activation of β-adrenergic receptors (β-ARs) by isoproterenol, through canonical and noncanonical (Gi and Gs Coupled) pathways forms LTP through activation of Ca^2+^/calmodulin-activated kinase-II, protein kinase A, and exchange protein activated by cAMP (Epac) which is critical for spine formation [93]. The α(2A)-adrenoreceptors (α(2A)-ARs) are highly expressed in neurons and essential for neuronal differentiation, growth, and neurotrophy. It was found that activation of α(2A)-ARs by guanfacine enhanced the expression of PSD-95, and significantly induced stubby and mushroom spines, along with enlargement of spine head size in cultured prefrontal cortex neurons. However, co-administration of yohimbine with guanfacin blocked the effects of guanfacine, confirming the role of α(2A)-ARs in the maturation of dendritic spines [94]. Furthermore, guanfacine prevented dendritic spine loss in layer II/III pyramidal neurons of prelimbic prefrontal cortex in rats exposed to chronic restraint stress [95]. In cultured cortical neurons from C57/B6 mice, guanfacine enhanced the expression of spinophilin (a key cytoskeletal protein involved in the formation and maintenance of dendritic spines) that leads to 1.2- and 1.8-fold increase in spine length and spine density, respectively [96]. PD rats treated with bromocriptine, a α-adrenergic agonist, showed significant increase in the number of synaptic contacts with dendritic spines compared to sham-operated rats indicating the indirect effect of bromocriptine on the memory and learning [97]. These findings collectively indicate that activation of adrenergic receptors have positive impact on the growth and distribution of dendritic spines.

### 3.6. Effect of Dopaminergic Agents on Dendritic Spines

Dopamine (DA) is implicated in several neuropsychiatric and neurological disorders as it regulates movement, motivation, reward, learning, and memory [98]. Primary prefrontal cortex (PFC) neuronal culture on repeated treatment with SKF81297, a DA agonist, resulted in increased dendritic branching and spine density, which is believed to be mediated through activation of D1 receptors and downstream signaling molecules (Rac1 and RhoA). While administration of SCH23390, a dopamine antagonist, reversed the SKF81297-induced increase in dendritic morphogenesis, indicating the positive role of D1 receptors in dendritic morphogenesis [99]. Increased spine density in hippocampal neurons is also reported by promoting transactivation of TrKB, mediated by D1 receptors [100]. D3 receptor agonists, ropinirole and pramipexole showed dose-dependent increase in the dendritic arborization in translational model of human-inducible pluripotent stem cells (hiPSCs) derived from dopaminergic neurons [101]. PD animal models have shown marked decrease in the number of spines and increase in spine head volume in striatal dendrites [102]. 6-hydroxydopamine-lesioned rats on chronic treatment with levodopa showed significant increase in the spine density and spine head area in the pyramidal tract neurons of primary motor cortex [103]. Further, the spine enlargement in the nucleus accumbens (NAc) and medium spiny neurons (MSNs) is linked to be associated with dopamine dysregulation syndrome (DDS). These observations are corroborated to dopaminergic degeneration and loss of dopamine in striatum and NAc [104]. Loss of nigrostriatal dopamine neurons in PD induces reduction in the number of dendritic spines with smaller cell body and less profusely arborized dendritic trees on MSNs of striatum expressing both D1 and D2 receptors [105]. However, non-selective activation of dopaminergic receptor by apomorphine (D1-D5 dopamine receptor agonist) resulted in learning and memory impairment, reduced dendritic length and LTP followed by neuronal damage in CA1 region of mice hippocampus [106]. Similar results were also observed in young Mongolian gerbil’s hippocampus on apomorphine treatment [107]. Another study revealed that brain-derived neurotrophic factor (BDNF)-induced chronic activation of D3 receptors improved motor functions and increased the dendritic spines in the striatal neurons of 6-hydroxydopamine (6-OHDA)-induced Parkinson’s rat [108].

Dopamine D(4) receptor activation upregulates the cofilin activity by dephosphorylating cofilin and thus depolymerizing actin [109]. 6-hydroxydopamine lesions in the ventral tegmental area disrupts the prefrontal cortex dopamine resulting in the decreased dendritic length and spine density of layer V pyramidal cells in the prelimbic cortex [110]. High-fat-diet-fed mice displayed reduced dendritic spine density in the substantia nigra due to neuroinflammation-mediated degeneration of dopaminergic neurons in midbrain [90]. Striatal dopamine loss is shown to decrease the number of MSN dendritic spines [111]. Activation of D1R and D2R receptors in a striatal cell culture containing MSNs increased the number of spines and spinophilin expression, indicating the direct role of these receptors on spines formation. Similarly, blockade of D1R and D2R receptors reduced the number of dendritic spines in cultured striatal MSNs indicating the role of dopamine in the spine formation [112]. It was also found that there was significant decrease in the association of spinophilin with neurofilament medium (NF-M) in dopamine-depleted striatum, causing decreased number of spines, as observed in Parkinson’s disease [113]. Thus, it is clear that spinophilin-induced alterations in spine density and morphology depends on D1 and D2 receptors which act as scaffolding key synaptic proteins [114]. Further, another study showed that alcohol-dependent rats fail to perform emotional learning tasks, while administration of L-DOPA during early withdrawal showed optimal learning along with increased number of long thin spines in MSNs of NAc. Another study revealed that drebrin, a dendritic spine protein depletion reduces D1 receptor levels and in turn reduces dendritic spine morphogenesis in mouse hippocampus [115]. These results display the role of dopamine in growth, maintenance, and restoration of spines [116].

The observations for all the neurotransmitter systems from various studies are summarized in Table 1.

## 4. Non-Therapeutic Modulators of Dendritic Spines

### 4.1. Impact of Calorie Restriction on Dendritic Spine

**Figure 2:**
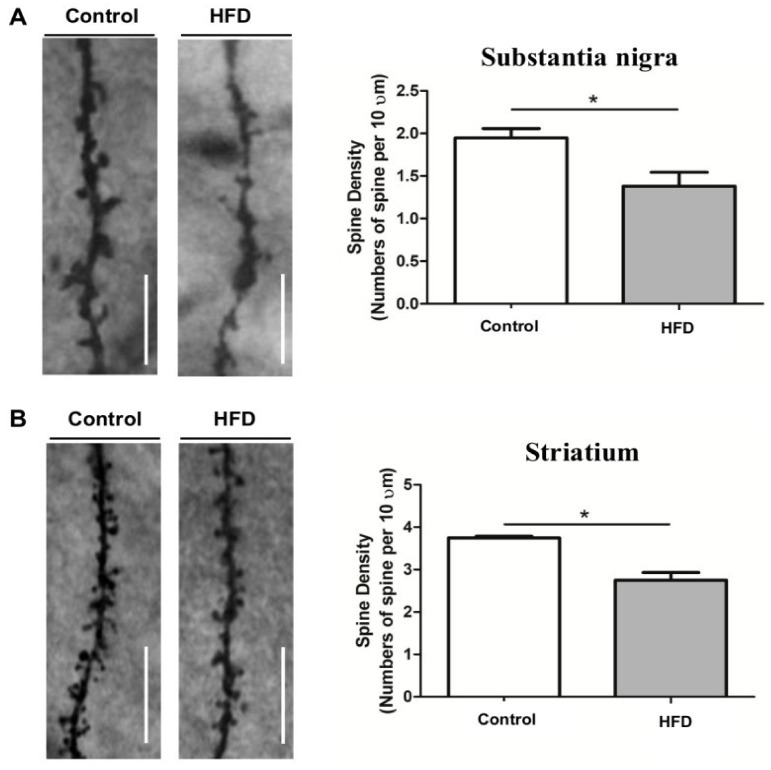
Representative Golgi staining images of dopaminergic neurons in substantia nigra and striatum [90] (reused as per the *International Journal of molecular Sciences* copyright permission policy). (**A**) Represents decreased dendritic spine density in substantia nigra of high fat diet (HFD) mice compared to control. (**B**) Represents decreased dendritic spine density in striatum of high fat diet (HFD) mice compared to control. * indicates the significant difference with respect to control.

Calorie restriction (CR) has shown prominent improvement in adult neurogenesis and neural plasticity [117,118], while high saturated fat diets have shown to impair learning and memory, demonstrated by decreased spine number and density in substantia nigra and striatum of mice brain (Figure 2) [90]. Diet that mimics fasting enhanced the hippocampal neurogenesis, upregulated neurogenic differentiation factor-1 (NeuroD1), spine density, and improved cognitive performance in aged mice [119]. Stranahan et al. (2009) investigated the effect of physical exercise and dietary restriction on hippocampal neuron morphology. Increase in neurotropin, BDNF, and density of dendritic spines was attributed to the dietary restriction, but not to the physical exercise. These data indicate that dietary restriction influences the biosynthesis of new dendritic spines and hence synaptic plasticity [120]. Rats fed ad libitum displayed significant loss of L-type spines (lollipop-shaped) in the basilar tree of layer V pyramidal cells of parietal cortex with normal ageing when compared to the diet-restricted rats [121]. Similarly, a reduction in dendritic spines density in CA1 region of hippocampus was observed along with insulin resistance in the high-fat-diet-fed rats [122]. Diabetic rats maintained on dietary restriction showed significant increase in the dendritic mushroom spines in the pyramidal neurons of CA1 region of hippocampus than diabetic rats fed ad libitum [123]. Further, mice maintained with CR and low protein-high carbohydrate (LPHC) diet showed increased dendritic spines in the dentate gyrus neurons [117].

Similarly, rats maintained with low-protein diets showed minimal decrease in the total number of neurons in dentate gyrus, CA1 and CA3 neurons compared to ad libitum fed rats. However, the feed-restricted rats also displayed reduced segments in the dendritic arborization of granule cells. However, there was an increase in the spine density in distal segments of dendritic spines and total number of axospinous synapses in dentate gyrus region. In addition, dendritic length of granule cells was also found to be increased. This study explains that controlled diet restriction improves the dendritic spines density in turn the synaptic functions [124].

On contrary, feed restriction in crucial stages of life viz feed restriction in new born rats between post-natal day 0 and 20 decreased dendritic spines in the layer V pyramidal cells in the frontal, parietal, and occipital cortices which may be related to the perinatal undernourishment [125]. Similarly post-mortem brains of severely malnourished infants showed short apical dendrites and fewer spines [126]. These data reveal that dietary interventions play critical role in dendritic spines formation and maintenance.

### 4.2. Effect of Pre-, Pro-, and Syn-Biotics on Dendritic Spines

Several studies have established the correlation between gut microbiota and neurological disorders including blood brain barrier integrity [127], neurogenesis [128], maturation, and ramification of microglia [129], myelination [130], expression of neurotrophins like BDNF, oxytocin, and vasopressin [131], and release of neurotransmitters [132]. Pre- and pro-biotics (text Box 1) are well-known for their positive influence on mental health, along with supporting the growth of commensal bacteria with psychophysiological effects, and hence they are also termed as psychobiotics [133,134]. The role of psychobiotics is not only limited to the regulation of neuroimmune axis and diseases of nervous system, but also involved the enhancement of cognition, and behavior phenomena [135].

The microbiome has attracted significant attention for its impact on the synaptic plasticity by modulating the dendritic spine morphology and distribution [136]. In light of this, germ-free (GF) animals have shown decreased dendritic size and fewer synaptic connections in the hippocampal pyramidal neurons. Indeed, absence of symbionts in mice was correlated to dendritic hypertrophy with more thin, stubby, and mushroom spines in the basolateral amygdala neurons, while the ventral hippocampal pyramidal neurons of GF mice displayed shorter, less branched spines with less stubby and mushroom types. These findings reveal a casual role of gut microbiota in the realm of spine pathology [136].

Multiple reports evidenced the involvement of BDNF in dendritic arborization and spine formation [137,138]. Moreover, administration of prebiotics and probiotics enhanced brain BDNF levels, which has prompted many researchers to examine the role of pre/probiotics on spine density and dendritic arborization in different diseased conditions [139,140]. Administration of antibiotics during pregnancy is reported to affect the maternal gut microbiota and alters the behavioral pattern in offspring [141].

Pregnant rats and their female offspring exposed to lead resulted in the loss of dendritic spines in both CA1 and dentate gyrus (DG) regions irrespective of the developmental stages (PND 22 or PND 68). Long-term intervention with multispecies probiotic (*Bifidobacterium longum* BL986, *Lactobacillus acidophilus* LA43, *Lactobacillus fermentum* LF26, *Lactobacillus helveticus* LH43, *Lactobacillus paracasei* LPC12, *Lactobacillus rhamnosus* LRH10, and *Streptococcus therophilus* ST30) restored the spine densities in both adolescence and adulthood [142].

In an in vivo study, high-fat-diet (HFD)-fed rats displayed hippocampal dysplasticity (decreased synaptic proteins like PSD-95, synaptophysin and spinophilin) including LTP impairment along with low grade local and systemic inflammation [143,144,145]. Earlier reports also suggest the evidence of gut dysbiosis on HFD consumption by enhancing the ratio of Firmicutes to Bacteroidetes ratio, thus promoting the growth of Proteobacteria [146,147]. On the other hand, decreased spine density in high-fat-diet-fed rats was restored on treatment with prebiotic Xyloolidosaccharide (XOS), probiotic *Lactobacillus paracasei* HIIO1 (*L. paracasei*) and synbiotics (combination of XOS and *L. paracasei*) [143]. Drebrin, a cytoplasmic actin-binding protein expressed in dendritic spines, regulates the morphology and plasticity of dendritic spines. Aberrant expression of drebrin indicates abnormal dendritic spines [148,149,150]. In an in vitro study, exposure of hippocampal neurons to live or heat inactivated *Lactobacillus rhamnosus* (LGG) and *Bifidobactererium bifidum* (TMC3115) increased the neuronal viability, and upregulated drebrin and SYP levels. Evidence strongly supports probiotics belonging to the family *Lactobacillus* and *Bifidobactererium* influence host neuronal development, brain biochemistry, and behavioral phenomenon [151].

1-methyl-4-phenyl-1,2,3,6-tetrahydropyridine (MPTP)-induced parkinsonism mice hippocampus showed increased expression of neuropsin, decreased expression of PSD-95 and synaptophysin along with reduced CA1 apical spine density. Administration of probiotic *Bifidobacterium breve* (MCC1274) reversed the MPTP-induced changes [152]. Aberrant higher induction of neuropsin in PD mice is linked to abnormal changes in the hippocampal plasticity [152]. Chronic administration of mixture of three *Bifidobacteria* (*B. longum*, *B. breve* and *B. infantis*) known as B-MIX to rats induced significant increase in thin and mushroom spine numbers in both apical and basal dendritic branches in CA1 region of hippocampus, while stubby spines remained unaltered. The mixture also positively enhanced the total dendritic length, total branch number, number of tips/neuron, and the number of pints/neurons, confirming the role of B-MIX mixture not only on spine density, but also on dendritic arborization [153].

### 4.3. Effect of Enriched Environment (EE) on Dendritic Spines

EE is well utilized experimentally to substantially mitigate conditions like depression and anxiety [154,155]. EE has shown to improve the rate of synaptogenesis and complex dendritic arbors formation [156]. Huntington’s disease (HD) mice exposed to EE showed the absence of dendritic spine pathology [157]. Maternal immune activation (MIA) of female rats downregulated HPA axis and glutamate signaling pathways in amygdala leading to alterations in behavior, endocrine status, and neural markers of synaptic plasticity [158]. MIA rats challenged with endotoxin lipopolysaccharide (LPS) during mid gestation (G15) induced alterations in the expression of genes critical to synaptic transmission and plasticity like Eaats, PSD-95, Gria1, and TrKB in amygdala, leading to the development of cognitive impairments. On postnatal day 50, the offspring were grouped to standard housing, communal nesting, and EE. The offspring exposed to EE rescued the deficits through reducing the activity of HPA axis, upregulating the synaptic markers like Eaat1, Eaat3, PSD-95, and TrKB, which otherwise reduced following prenatal immune activation. The upregulation in synaptic markers indirectly indicate the enhancement of dendritic spines [159]. SNK-SPAR pathway is involved in activity-dependent remodeling of synapses [160]. Similarly, studies have illustrated the mechanism of NWASP-ARP2/3 pathway in regulating spine and synapse formation via ARP2/3-mediated branching of actin for dendritic head enlargement and maturation [161]. Pregnant rats exposed to heavy metal mixtures (MM) via drinking water throughout gestation (from G0) and the offspring were continued to receive the MM throughout lactation, and were experimentally grouped into control and EE-treated groups. The offspring from control group showed impaired cognitive performance correlated to the decreased dendritic spine density, decreased number of mushroom type spines, activation of SNK-SPAR, and inhibition of NWASP-ARP2/3 pathways. However, EE-treated offspring reversed MM-induced disruptions [162]. Few studies claim the necessity of zinc for experience-dependent plasticity in brain. A study conducted by McAllister et al. reported the beneficial effects of EE are independent of zinc in terms of increased basal dendritic length and spine density in neurons of barrel cortex, striatum and amygdala [163]. Amyloid beta (Aβ) oligomer, a synaptotoxic agent, induced the impairment of LTP through downregulation of miRNA-132 expression and increased histone deacetylase (HDAC3), while EE produced beneficial effects through suppressing the effects of Aβ oligomer in hippocampus of wild-type mice [164].

In mice subjected to cerebral focal ischemia by occluding middle cerebral artery, exposure to EE increased the synapse numbers along with the upregulation of expression of synaptophysin and GAP-43, indicating the important role of EE in promoting synaptic remodeling [165]. Recognition memory impairment induced by MK-801, a NMDA antagonist was significantly reversed by EE through restoration of LTP and dendritic spines in long Evans rats [166]. Deafening protocol in rats significantly decreased apical and basal spine density indicating the absence of auditory stimulation induced neuronal atrophy in auditory cortex. Auditory environmental enrichment increased the number of action potentials recorded and glutamergic synapses in layer II/III of auditory cortex along with increase in apical dendritic length and spine density in pyramidal neurons of auditory cortex [167].

### 4.4. Effects of Yoga and Meditation on Dendritic Spine

Yoga and meditation offer several health benefits including enhanced memory, emotional and attention control, improvement in depressive, anxious, and stressful behavior [168,169,170]. However, the effects of yoga and meditation on central nervous system function still need to be studied more scientifically. Recently, Villemure et al. (2021) reported that yogic practitioners have greater grey matter volume compared to non-yogic practitioners [171]. Though many studies proved the beneficial role of yoga and meditation practice on memory and cognition, not much information is available on their impact on dendritic spines. Practice of Hatha yoga is corroborated to the increased grey mater volumes in the frontal, limbic, temporal, occipital, and cerebellar regions and improved performance in cognitive failure questionnaire which is linked to the neuroplastic changes in the brain [172]. Sahaja yoga facilitates emotional and attentional control which is again linked to the neuroplastic changes in the temporal and frontal areas of cerebrum [173]. Intriguingly, the larger grey matter volume, evidenced from magnetic resonance imaging, in long-term mediators is linked to the improved cognitive, emotional, and attention control [174]. Mounting evidence shed light on the benefits of different yoga and meditation techniques on the cognition, emotional balance, attention control, reward, and happiness which is correlated to increased grey matter volume [174,175,176,177,178]. Indeed, the loss of dendritic spines is positively correlated to decrease in grey matter volume in stressful conditions in mice [179]. Thus, the increased grey matter volume in yoga and meditators might be due to the positive changes in the dendritic spine numbers/density. However, imaging the morphology and distribution of dendritic spines in yoga and meditators will provide interesting evidence and studies are warranted in this line.

Box 1 **Definitions.**

**Dendritic Spine sprouting**: “Recurving of distal dendritic arbors, short-segment branching, growth cone-like processes initiating at the neuronal dendritic process is spine sprouting” [39].
**Siddha medicine**: “Siddha is an ancient traditional Indian system of medicine practiced in southern part of India. Siddha medicine is claimed to revitalize and rejuvenate dysfunctional organs that cause the disease. Kayakarpam, a special combination of medicine and life style, Varmam therapy, Vaasi (Pranayamam) and Muppu the universal Salt are the specialties of Siddha system of medicine. Thus, this system connects both spiritual and physical and treats the person as a whole i.e., it concentrates on the physical, psychological, social, and spiritual wellbeing of an individual” [180].
**Prebiotics**: “Are the source of food for gut’s healthy bacteria or the food that induces growth or activity of beneficial microorganisms in the gut”.
**Probiotics**: “Are the live microorganisms, when consumed improve or restore the gut flora”.
**Synbiotics**: “Are mixture of both prebiotics and probiotics viz, fermented food that synergistically improve the gut flora” [181].

## 5. Conclusions

Spinogenesis has a functional role in memory formation and retrieval, and is regulated by multiple factors like PAK, CaMKII, syndecan-2, Ena/VASP, paralemmin-1, TLCN, PSD, AF6/afadin, Kalirin-7, Rac1, Semaphorin 3F, Tiam1-Rac1-3-LIMK1/2-Cofilin1, and RhoA-ROCK1/2-Myosin II pathways. Autism spectrum disorder (ASD), schizophrenia, and AD are the neurological disorders characterized by significant disruptions in processing information and cognition. Although multiple studies suggest synaptic dysfunctions as a leading insult to neuronal death, disease-specific disruptions in spine shape, size, or number accompany a large number of brain disorders, suggesting dendritic spines as target for many neurodegenerative disorders. Exploration of relationship between dendritic spines and neurological diseases unlocked spine dysmorphogenesis as one of the main underlying causes. This led to the identification of new windows of opportunities for therapeutic intervention.

**Figure 3:**
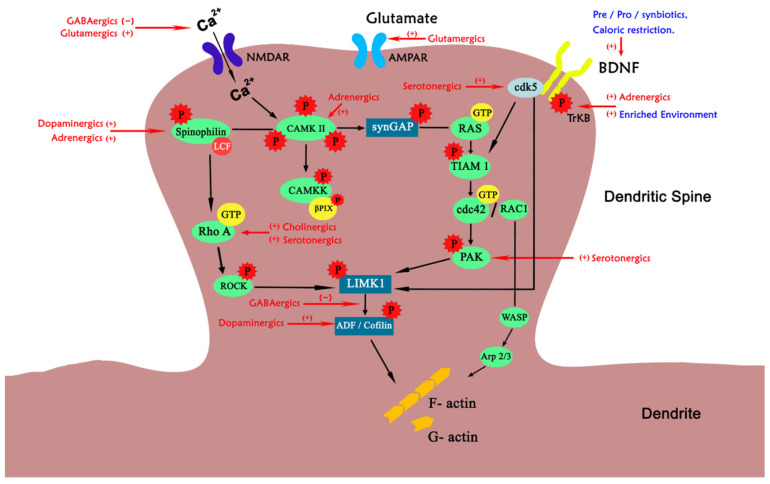
Pharmacological and non-pharmacological targets on structural pathways in dendritic spine. In the figure, the descriptions in red colour indicates pharmacological modulators and blue indicates non-pharmacological modulators on various phases of dendritic spine morphogenesis. (+) denotes positive modulation; (−) indicates negative modulation. NMDAR: N-methyl-D-aspartate receptor, AMPAR: α-amino-3-hydroxy-5-methyl-4-isoxazolepropionic acid receptor, cdK5: Cyclin dependent kinase-5, BDNF: Brain derived neurotrophic factor, CaMKII: Calcium/calmodulin-dependent protein kinase II, TrKB: Tropomyosin receptor kinase-B, TIAM1: T-cell lymphoma invasion and metastasis-inducing protein 1, cdc42: Cell division control protein 42, WASP: Wiskott-Aldrich syndrome protein.

Experimental studies have demonstrated that application of neurotransmitters and chemical messengers like acetylcholine, adrenaline, dopamine, glutamate, GABA, and 5-HT elicits alterations in spine structure and functions (Figure 3). Incidentally, alteration in these chemical messengers is also one of the underpinning pathological mechanisms for most of the neurological disorders. These chemical messengers have been reported to be involved in rectifying both structural and distributional pathologies of dendritic spines and hence can be of therapeutic interest. Interestingly, various studies have shown the role of life style modifications like reduced calorie intake, inclusion of pro/pre/synbiotics in the diet, exposure to enriched environment, and regular practice of yoga and meditation in improving the dendritic spine pathologies as shown in Figure 3 As per the observations from this review it can be proposed that effect of non-pharmacological interventions on the dendritic spine morphogenesis is on par with the pharmacological interventions. Thus, non-pharmacological interventions can be advised as supplementary to neurodegenerative diseases associated with cognitive decline.

Overall, this review robustly reinforces the application of non-pharmacological modulators to treat spine pathologies as an appropriate approach. However, new discoveries that contribute to the elucidation of beneficial effects of therapeutic vs. non-therapeutic modulators are much needed. More detailed mechanistic studies on non-therapeutic modulators are imperative because the non-therapeutic interventions may allow the development of newer avenues in treating spine pathologies and thus the underlying neurological diseases.

## 6. Limitations

In the present review, an attempt is made to collate information that demonstrate the influence of various neurotransmitter systems, chemical messengers, and non-pharmacological approaches on the structural and functional status of dendritic spines. Majority of the evidence on the effects of neurotransmitters on the dendritic spines are obtained from preclinical studies, while only a few clinical data are available, even in recent years. Further, in many instances the results of the in vivo studies are not specific, as the effects observed may also be contributed by the overlapping role of other neurochemical systems. Hence, specific and elaborate in vitro studies are needed. Although preliminary research data have provided significant evidence on improved cognitive performance following non-pharmacological approaches, by altering the dendritic spine turnover and shape, only limited literature are available on the spine turnover, and imaging-based structural information before and after the intervention.

These findings underpin the urgent need for intense preclinical and clinical investigations on both pharmacological and non-pharmacological modulators, the combination of which may impart synergistic effects on dendritic spines, in turn cognitive performance, and shall be of transitional importance.

## Figures and Tables

**Table 1:** Effect of various neurochemical systems on dendritic spines.

Sl. No	Neurochemical System	Drug/Chemical Treatment	Effect on DS						References
			Number	Density	Length	Size	Morphogenesis	Enlargement	
1	Cholinergic	Donepezil, LM11A-3, Brahmi Nei, Phenserine tartarate, Huperzine A,	**↑**	**↑**	**↑**	-	-	-	[53,55,56,57,58]
2	Glutamatergic	Riluzole, Ketamine, LY341495	**↑**	**↑**	-	-	-	-	[69,72,73]
3	GABAergic	Bicuculline, mercaptopropionic acid	-	**↑**	-	-	-	-	[79]
4	Serotonergic	LP-211, Olanzapine, Vortioxetine,	**↑**	**↑**	-	**↑**	**↑**	**↑**	[84,87,88]
5	Adrenergic	Guanfacine, Bromocriptine, isoproterenol	-	**↑**	**↑**	**↑**	-	-	[93,94,97]
6	Dopaminergic	SKF81297, Ropinirole, Pramipexole	**↑**	**↑**	**↑**	-	**↑**	-	[99,101]

**↑** denotes increase; - denotes no information.

## Data Availability

All the literature collected from search engines, journal prints, printouts related copyright permission for reuse of figures etc., are available in the IT section of SBC lab.
